# A Challenging Case of Isolated Pulmonic Valve Endocarditis With Septic Embolic and High-Grade Bacteremia

**DOI:** 10.7759/cureus.72007

**Published:** 2024-10-21

**Authors:** Miguel Felix, Antonio Diaz Lizarraga, Melanie Diaz Ortiz, Pedro Arthur Macedo de Freitas, Thomas Treadwell

**Affiliations:** 1 Department of Medicine, MetroWest Medical Center, Framingham, USA

**Keywords:** iv drug abuser, mssa bacteremia, pulmonary valve replacement, pulmonic valve endocarditis, septic pulmonary emboli

## Abstract

Isolated pulmonic valve endocarditis is a rare entity attributed to multiple factors, including lower pressures on the right side of the heart, as well as lower oxygen content of venous blood. Herein, we present a challenging case of isolated pulmonic valve endocarditis complicated with septic emboli and high-grade methicillin-sensitive Staphylococcus aureus (MSSA) bacteremia. A 31-year-old female presented to the emergency department with fever, chills, fatigue, left-sided pleuritic pain, shortness of breath, and an 8-pound weight loss for the past two weeks. She is an active intravenous drug user. Notable was a grade four diastolic murmur most prominent on the upper left sternal border. Chest CT with contrast showed multiple pulmonary emboli with cavitating nodules suspicious of a septic etiology. Transthoracic echo revealed an isolated large irregularly shaped (3.0 cm x 1.5 cm) vegetation on the pulmonic valve with moderate-to-severe pulmonary regurgitation. The course was notable for persistent high-grade MSSA bacteremia for a total of 10 days, which was treated with ertapenem and cefazolin. The patient did not undergo valve replacement. Close outpatient follow-up was established with a recommendation to treat with an additional two doses of dalbavancin 1,500 mg one week apart. Isolated pulmonic valve endocarditis is a very rare entity. Prompt recognition of isolated pulmonic valve endocarditis and multidisciplinary management is key to improving outcomes among patients with this extremely rare condition.

## Introduction

Pulmonic valve (PV) endocarditis is considered a very rare entity, accounting for 1.5%-2% of total cases of infective endocarditis [1]. Its rarity has been attributed to multiple factors, including lower pressures on the right side of the heart, as well as lower oxygen content of venous blood. In regards to the risk factors, most events of PV endocarditis occur in individuals with a history of IV drug abuse, which relates to this case report. Additionally, other circumstances have been associated with PV endocarditis: central venous catheters, dental procedures, alcoholism, organ transplants, bowel surgery, congenital heart disease, immunosuppressive therapy, and gonorrhea.

Literature on pulmonic valve endocarditis is relatively scant, but cases are often complex due to the high risk of complications, including septic emboli and pulmonary hemorrhage. Diagnosing PV endocarditis is also a challenge because is sometimes difficult to visualize the pulmonic valve clearly. The pulmonic valve's location, being the most forward and farthest from the ultrasound probe compared to other heart valves, makes its visualization challenging [2].

Herein, we present a challenging case of isolated pulmonic valve endocarditis complicated with septic emboli and high-grade methicillin-sensitive Staphylococcus aureus (MSSA) bacteremia.

## Case presentation

A 31-year-old female presented to the emergency department with fever, chills, fatigue, left-sided pleuritic pain, shortness of breath, and an 8-pound weight loss for the past two weeks. She is an active intravenous drug user.

Upon examination appears cachectic and ill, with an excoriated rash on both forearms. Notable was a grade 4 diastolic murmur most prominent on the upper left sternal border. She did not exhibit peripheral edema, ascites, or jugular venous distention. Detailed laboratory values are shown in Table 1.

**Table 1:** Selected initial laboratory values. Bolded numbers represent out-of-range values.

Parameter	Value	Reference range
WBC	8.2	4-11.0 × 10^9^/L
Hemoglobin	9	12-16 g/dL
Platelets	87	150-450 × 10^9^/L
Lactic acid	1.4	<2 mmol/L
Pro-BNP	996	0-450 pg/mL
Troponin T	6	<14 ng/L
Procalcitonin	10.08	<0.08 μg/L
Urine toxicology	+ cocaine, fentanyl, oxycodone	N/A

**Figure 1:**
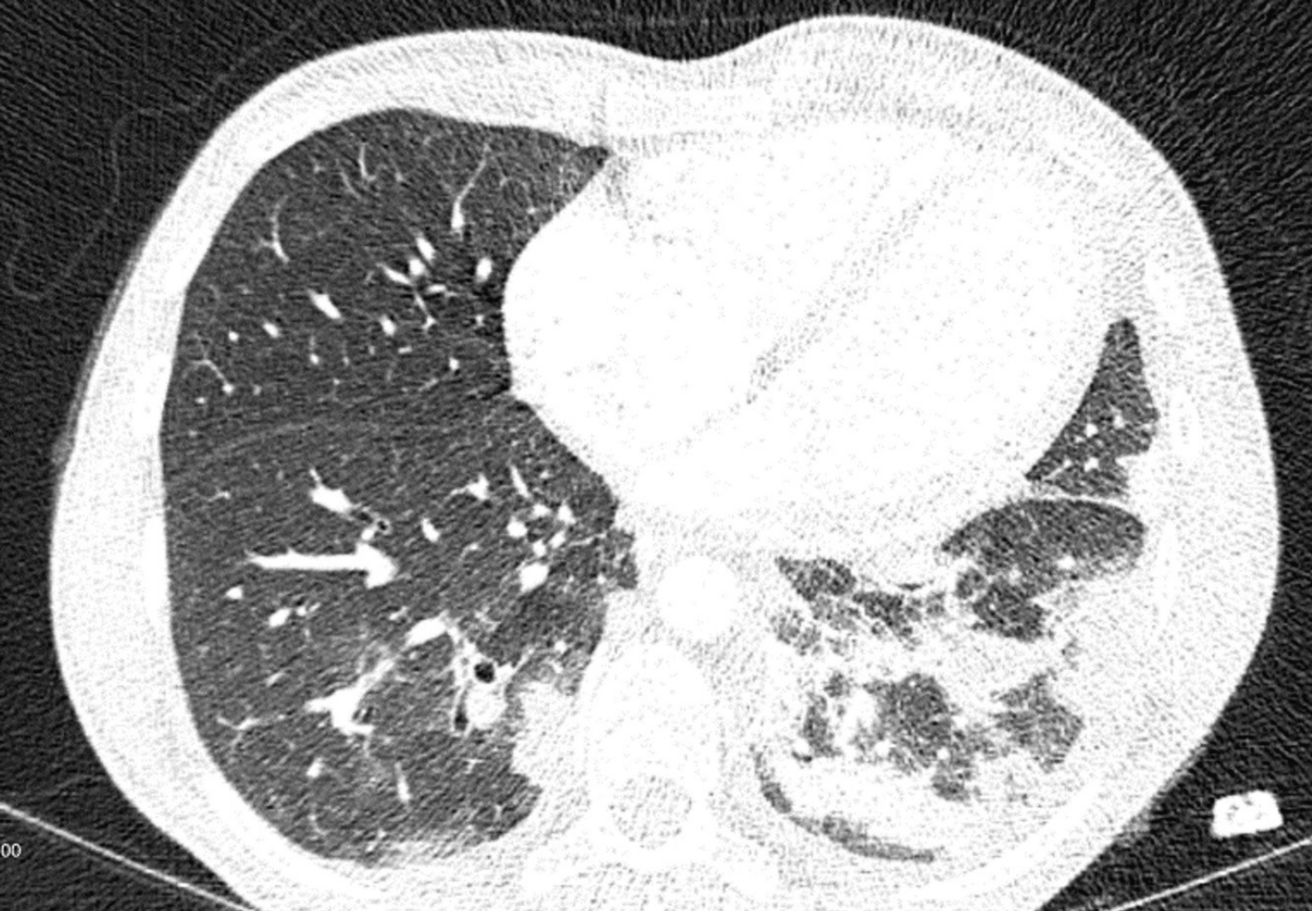
CT of the chest demonstrating moderate-to-large embolic load bilaterally with scattered peripheral nodular densities and consolidations.

**Figure 2:**
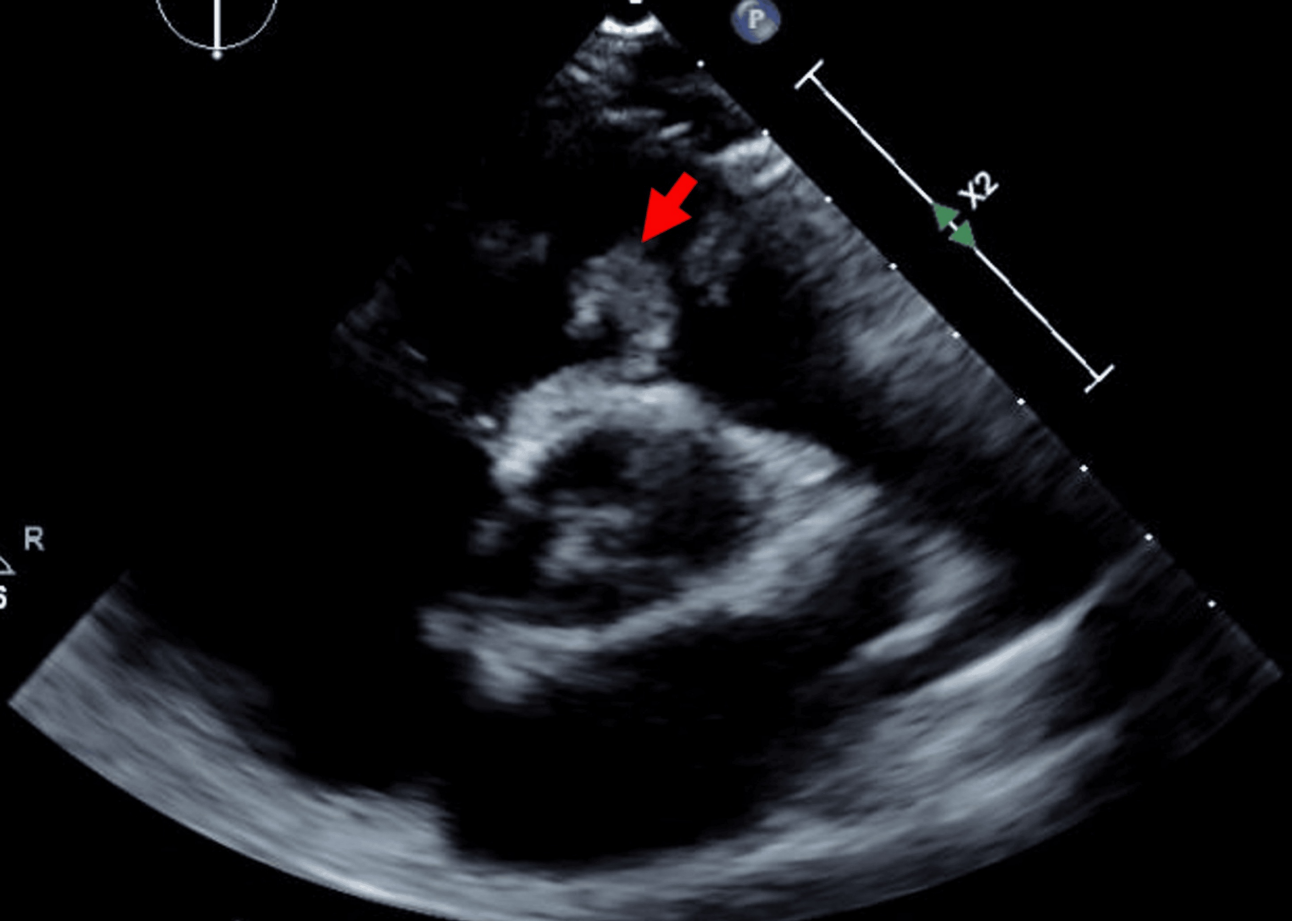
Parasternal short-axis view showing an isolated large irregularly shaped (3.0 cm x 1.5 cm) mass on the pulmonic valve consistent with vegetation. The red arrow depicts the vegetation.

Chest CT with contrast showed multiple pulmonary emboli with cavitating nodules suspicious of a septic etiology (Figure 1). Transthoracic echo revealed an isolated large irregularly shaped (3.0 cm x 1.5 cm) mass on the pulmonic valve consistent with vegetation (Figure 2). There was also moderate to severe pulmonary regurgitation. No evidence of right-sided heart strain. 

The patient was started on therapeutic anticoagulation for pulmonary embolism, as well as vancomycin. Shortly after blood cultures started to grow gram-positive cocci in clusters. Transfer to a tertiary care center was initiated due to the need for cardiothoracic evaluation and potential hemorrhagic complications of anticoagulation.

Tertiary care center course notable for persistent high-grade MSSA bacteremia for a total of 10 days. Antibiotics were switched to cefazolin and ertapenem. She was not deemed an ideal cardiac surgical candidate given her IV drug use. Anticoagulation was stopped.

The patient had a good clinical response to treatment as the fever resolved, bacteremia cleared, and, more surprisingly, repeat transthoracic echocardiogram upon discharge showed resolution of vegetation. Close outpatient follow-up was established with a recommendation to treat with an additional two doses of dalbavancin 1,500 mg one week apart.

## Discussion

Isolated pulmonic valve endocarditis is a very rare entity. A previous case series from a tertiary center in India found eight cases within a 28-year period [2]. Most of them had prolonged fever and received at least two to three weeks of antibiotics. Similarly, our patient presented with several weeks of fever and required prolonged antibiotic therapy to clear the bacteremia. Interestingly, she lacked signs of heart failure, as opposed to 50% of the cases reported in that case series.

Risk factors include congenital cardiac abnormalities, implanted catheters or devices, and intravenous drug use [3]. The latter appears to be a significant factor in its development. Our patient was an active IV drug user, and this had significant implications for both inpatient management and follow-up. For this reason, she was not deemed an ideal surgical candidate, and she was not discharged with indwelling intravascular access. This seems to be a relatively common practice in major centers for a few reasons. In the absence of treatment for substance use disorder, patients with active IV drug use have a higher risk of recurrent endocarditis likely due to recurrent drug use, a higher probability of death in the year after the operation as compared to patients that do not use IV drugs, and more complex cardiac involvement requiring long stays in the intensive care unit and hospital postoperatively. This represents a significant burden, particularly among smaller hospitals since current risk models do not accurately adjust for known comorbidities such as hepatic dysfunction, abscess and fistula formation, and other mental health issues such as increased suicide risk, which are far more common among IV drug users [4].

Currently in the US, injection drug use is a major risk factor for endocarditis complicating both inpatient and outpatient management. Our patient was transferred because of the possible need for surgery, but cardiac surgeons are reluctant to replace valves in these patients because of higher probabilities of death and reinfection following valve surgery. Inpatient stays are often complicated by metastatic infection, prolonged bacteremia, and struggles with the management of withdrawal and pain. Outpatient parenteral antibiotics are usually not done because of concerns of misuse of catheters for illicit drug use, and, increasingly, antibiotic regimens include oral agents, not the standard of care in this country.

Surgical indications for infective endocarditis generally include signs of severe heart failure or valvular dysfunction, disease in a prosthetic valve, infection beyond the valve leaflets, recurrent systemic embolization, large mobile vegetations (typically greater than 1 cm for pulmonic valve endocarditis with pulmonic septic emboli), or persistent sepsis despite adequate antibiotic therapy for more than five to seven days [5].

## Conclusions

Isolated pulmonic valve endocarditis is a very rare entity accounting for a minority of infectious endocarditis cases. In this case, the patient responded well to treatment and had good follow up after discharge. This outcome is in part to the early recognition of her diagnosis in the community hospital with prompt transfer to tertiary care center for multidisciplinary management. Prompt recognition and multidisciplinary management is essential to prevent complications and improve outcomes.
